# Income Level and Impaired Kidney Function Among Working Adults in Japan

**DOI:** 10.1001/jamahealthforum.2023.5445

**Published:** 2024-03-01

**Authors:** Nana Ishimura, Kosuke Inoue, Shiko Maruyama, Sayaka Nakamura, Naoki Kondo

**Affiliations:** 1 Department of Social Epidemiology, Graduate School of Medicine, Kyoto University, Kyoto, Japan; 2 Hakubi Center, Kyoto University, Kyoto, Japan; 3 Institute for Economic and Social Research, Jinan University, Guangzhou, China; 4 Department of Economics, Sophia University, Tokyo, Japan

## Abstract

**Importance:**

Chronic kidney disease (CKD) is a major public health issue, affecting 850 million people worldwide. Although previous studies have shown the association between socioeconomic status and CKD, little is known about whether this association exists in countries such as Japan where universal health coverage has been mostly achieved.

**Objective:**

To identify any association of income-based disparity with development of impaired kidney function among the working population of Japan.

**Design, Setting, and Participants:**

This was a nationwide retrospective cohort study of adults aged 34 to 74 years who were enrolled in the Japan Health Insurance Association insurance program, which covers approximately 40% of the working-age population (30 million enrollees) in Japan. Participants whose estimated glomerular filtration rate (eGFR) had been measured at least twice from 2015 to 2022 were included in the analysis, which was conducted from September 1, 2021, to March 31, 2023.

**Exposure:**

Individual income levels (deciles) in the fiscal year 2015.

**Main Outcomes and Measures:**

Odds ratios were calculated for rapid CKD progression (defined as an annual eGFR decline of more than 5 mL/min/1.73 m^2^), and hazard ratios, for the initiation of kidney replacement therapy (dialysis or kidney transplant) by income level deciles in the fiscal year 2015.

**Results:**

The study population totaled 5 591 060 individuals (mean [SD] age, 49.2 [9.3] years) of whom 33.4% were female. After adjusting for potential confounders, the lowest income decile (lowest 10th percentile) demonstrated a greater risk of rapid CKD progression (adjusted odds ratio, 1.70; 95% CI, 1.67-1.73) and a greater risk of kidney replacement therapy initiation (adjusted hazard ratio, 1.65; 95% CI, 1.47-1.86) compared with the highest income decile (top 10th percentile). A negative monotonic association was more pronounced among males and individuals without diabetes and was observed in individuals with early (CKD stage 1-2) and advanced (CKD stage 3-5) disease.

**Conclusions and Relevance:**

The findings of this retrospective cohort study suggest that, even in countries with universal health coverage, there may be a large income-based disparity in the risk of rapid CKD progression and initiation of kidney replacement therapy. These findings highlight the importance of adapting CKD prevention and management strategies according to an individual’s socioeconomic status, even when basic health care services are financially guaranteed.

## Key Points

**Question:**

Is there an association between income levels and the development of impaired kidney function among the working population in a country with an established universal health care system?

**Findings:**

This retrospective cohort study of 5.6 million adults found that those in the lowest compared with the highest income decile showed increased risk of rapid chronic kidney disease (CKD) progression and kidney replacement therapy initiation. A negative monotonic association with rapid CKD progression was more evident among males and individuals without diabetes.

**Meaning:**

These findings indicate that income-based disparities are associated with the development of impaired kidney function in the context of universal health care, highlighting the crucial role of comprehensive CKD prevention and management strategies for low-income workers.

## Teaser

This cohort study investigates the association of income-based disparity with impaired kidney function among a working population in a country with universal health care.

## Introduction

Chronic kidney disease (CKD) is a major public health issue, affecting 850 million people worldwide.^1,2^ Consistent with the rapid increase in the prevalence and complications of CKD during recent decades,^1,2^ it is projected to become the fifth most common cause of years of life lost by 2040.^3^ CKD causes severe long-term health consequences, including cardiovascular disease, mortality, and reduced quality of life through physical and psychological dysfunction.^4,5,6,7,8^ In addition, once patients develop end-stage kidney disease, kidney replacement therapies (KRTs)—ie, dialysis and kidney transplant—are required and often carry catastrophic medical costs.^9^ Therefore, it is imperative to prevent CKD to improve population health and ensure the sustainability of health care systems.

Despite the substantial attention to the role of socioeconomic status (eg, income, educational attainment, and residential area) in the development and progression of CKD,^10,11,12,13,14,15,16,17,18,19,20,21^ health disparities associated with socioeconomic factors in CKD have not been reduced worldwide.^22,23^ This dearth of progress may apply to countries with universal health coverage, such as Japan. A recent longitudinal study showed that disparities in other cardiovascular risk factors (eg, obesity, hypertension, and diabetes) by individual income levels exist, or are even increasing among the working-age population in Japan.^24^ However, evidence is limited regarding the extent of income-related disparities associated with CKD in countries with universal health coverage. Moreover, to our knowledge, no previous nationwide study has assessed the decline volume in the estimated glomerular filtration rate (eGFR) by individual income level.

The Japanese health system mandates annual health examinations for all citizens and ensures patients have free access to medical services, with no gatekeeping regulations for selecting medical care facilities. These programs could have the potential to reduce health disparities; therefore, benchmarking the levels of income-based disparity in medical outcomes related to CKD in this population could advance our understanding of the socioeconomic status (SES) disparities in CKD, and provide clinicians and decision-makers with the crucial information needed to combat CKD. Given the difference in income levels by sex and difference in the risk of developing or progressing CKD according to diabetes status and baseline kidney function,^25,26,27^ we also hypothesized that there would be heterogeneity in the income-CKD association according to these baseline characteristics. Therefore, considering these various factors, we sought to assess the association between individual income and subsequent rapid CKD progression and initiation of KRT in the context of universal health coverage.

## Methods

This study was approved by the institutional review board of Kyoto University (approval number R3021), which waived the requirement for informed consent owing to the use of anonymized data. This study followed the Strengthening the Reporting of Observational Studies in Epidemiology (STROBE) reporting guideline.

### Data Source and Participants

We used the nationwide database managed by the Japan Health Insurance Association, which provides health insurance to employees of medium- and small-sized enterprises and their dependents. The database includes records of medical claims, health examinations, and basic sociodemographic information (additional information on this database is provided in eMethods 1 in Supplement 1).

First, we identified 17 990 680 insured individuals (excluding dependents) aged 35 to 74 years (eligible ages for specific health examinations for insured individuals in the Japan Health Insurance Association) in the fiscal year 2015 (April 1, 2015-March 31, 2016). Second, we extracted 7 512 083 individuals who underwent a health examination in the fiscal year 2015 (defined as baseline examination). Third, we excluded individuals receiving dialysis, including hemodialysis or peritoneal dialysis, until 1 month after the baseline examination; pregnant females, based either on code O00–99 from the *International Statistical Classification of Diseases and Related Health Problems, Tenth Revision (ICD-10)*, or the records of childbirth allowance payment; those lacking kidney function measurements on or after the baseline examination; and individuals with missing data on covariates at baseline. We also excluded individuals with outliers on eGFR values (>99.9 percentile for each year) or covariates, including body mass index (BMI, calculated as weight in kilograms divided by height in meters squared), waist circumference, hemoglobin (Hb), systolic blood pressure (SBP), low-density lipoprotein cholesterol (LDL-C), high-density lipoprotein cholesterol (HDL-C), triglycerides, blood glucose, and uric acid (>99.9 percentile or <0.1 percentile). In all, 5 591 060 individuals were included in the analysis (eFigure 1 in Supplement 1). Of these, 82.5% were followed up for more than 3 years, 69.0% for more than 5 years, and 61.4% until the end of the study period.

### Measurement of Income Level

Individual income was constructed based on salaries for the fiscal year 2015 collected from insurance premium information. The income data were reported by 50 categories, with level 1 indicating $5032 (¥696 000) per year and level 50 indicating $120 590 (¥16 680 000) per year. We categorized individuals into income deciles, which served as our primary exposure groups. To convert the Japanese yen to US dollars, we used the rate on July 12, 2023, which was $1 equivalent to ¥138.32.

### Outcomes

Two outcomes related to the progression of kidney dysfunction were evaluated: (1) rapid CKD progression (defined as an annual eGFR decline of more than 5 mL/min/1.73 m^2^)^28^ and (2) initiation of KRT, defined as the first record of continuous dialysis (hemodialysis or peritoneal dialysis) or kidney transplant (living donor or cadaveric kidney transplant), 1 month after a physical examination in the medical claims data. Details on eGFR calculation are described in eMethods 2 in Supplement 1.

### Other Covariates

The covariates include sociodemographic factors, smoking habits, physical measurements, and results of other laboratory tests at the baseline examination. Smoking-related information was obtained at the examinations. We evaluated comorbidities (hypertension [I10–15], diabetes [E10, E11], cardiovascular disease [I20–69], cancer [C00–97], dyslipidemia [E78.8, E78.9], and hyperuricemia [E79.0]) based on *ICD-10* codes. Comorbidities were determined using diagnostic codes in insurance claims during the fiscal year 2015.

### Statistical Analysis

To investigate the association between income and each outcome, we used 2 models: (1) logistic regression models for rapid CKD progression and (2) Cox proportional hazards regression models for initiation of KRT. We also used linear regression models for the secondary outcome of annual eGFR decline volume. All models were adjusted for age, sex, smoking status, BMI, waist circumference, Hb, SBP, LDL-C, HDL-C, triglycerides, blood glucose, uric acid, and the stated comorbidities. Given that standard income levels vary by area of residence, we included prefecture fixed effect (n = 47). We evaluated the validity of the proportional hazard assumption for the Cox proportional hazards regression model using Schoenfeld residuals.^29^ Furthermore, we conducted subgroup analyses by sex (female and male), diabetes status (diabetes and nondiabetes), and baseline CKD stage (early stage, 1-2; advanced stage, 3-5). For the subgroup analyses by sex, we used sex-specific income deciles because income distribution significantly differed by sex. Because each subgroup differed in the effects of the covariates, we tested for interaction using the Bland-Altman method.^30^ We also conducted several sensitivity analyses and additional analyses as described in eMethods 3 and 4 in Supplement 1.

Statistical tests were 2-tailed and *P* values < .05 were considered statistically significant. Data analyses were conducted from September 1, 2021, to March 31, 2023, using Stata, release 17.0 (StataCorp LLC) and the Health Disparities Calculator, version 2.0.0 (US National Cancer Institute).^31,32^

## Results

The mean (SD) age of the 5 591 060 individuals was 49.2 (9.3) years; females composed 33.4% and males 66.6% of the group. Females constituted 71.1% of the lowest income group, and males constituted 90.7% of the highest income group (Table; eTable 1 in Supplement 1). The highest and lowest income groups showed a higher prevalence of older individuals and comorbidities and lower eGFR, compared with the means; 93.7% had a normal range of kidney function (ie, eGFR ≥60 mL/min/1.73 m^2^). The overall prevalence of hypertension and diabetes was lower than that of the general Japanese population.^33^ The sex-specific distribution of covariates by income level is available in eTables 2 and 3 in Supplement 1. Compared with individuals without health examination data in 2015, those included in this study were more likely to be male and have a higher income level (eTable 4 in Supplement 1).

**Table.** Characteristics of the Study Population, by Individual Income Level

Characteristic	Overall	Income deciles	
		Decile 1-5 (≤median)	Decile 6-10 (>median)
Total participants, No.	5 591 060	2 846 136	2 744 924
Income, mean (SD), $/y	28 102 (19 659)	17 897 (4454)	38 684 (23 381)
Age, mean (SD), y	49.2 (9.3)	50.3 (9.9)	48.1 (8.4)
Sex, No. (%)			
Female	1 867 991 (33.4)	1 478 634 (52.0)	389 357 (14.2)
Male	3 723 069 (66.6)	1 67 502 (48.0)	2 355 567 (85.8)
Smoking, No. (%)	1 921 317 (34.4)	847 620 (29.8)	1 073 697 (39.1)
BMI, mean (SD)	23.3 (3.7)	23.0 (3.8)	23.7 (3.5)
Waist circumference, mean (SD), cm	82.5 (9.9)	81.3 (10.2)	83.9 (9.5)
eGFR, mean (SD), mL/min/1.73 m^2^	79.7 (14.2)	80.1 (14.7)	79.4 (13.7)
CKD stage (eGFR, mL/min/1.73 m^2^), No. (%)			
1 (≥90)	1 210 444 (21.7)	653 188 (23.0)	557 256 (20.3)
2 (60-89)	4 025 783 (72.0)	2 003 766 (70.4)	2 022 017 (73.7)
3 (30-59)	347 096 (6.2)	184 694 (6.5)	162 402 (5.9)
4 (15-29)	4183 (0.07)	2418 (0.08)	1765 (0.06)
5 (<15)	3554 (0.06)	2070 (0.07)	1484 (0.05)
Hemoglobin, mean (SD), g/dL	14.5 (1.5)	14.1 (1.6)	14.9 (1.3)
Systolic BP, mean (SD), mmHg	122.4 (16.9)	122.3 (17.5)	122.5 (16.2)
LDL-C, mean (SD), mg/dL	124.3 (31.5)	123.2 (31.5)	125.5 (31.4)
HDL-C, mean (SD), mg/dL	61.9 (16.5)	64.7 (16.9)	59.1 (15.6)
Triglycerides, mean (SD), mg/dL	113.5 (85.6)	103.0 (76.1)	124.5 (93.1)
Glucose, mean (SD), mg/dL	97.6 (19.0)	96.8 (18.7)	98.5 (19.2)
Uric acid, mean (SD), mg/dL	5.6 (1.4)	5.2 (1.4)	5.9 (1.3)
Comorbidity, No. (%)			
Cancer	145 893 (2.6)	87 403 (3.1)	58 490 (2.1)
Cardiovascular disease	472 165 (8.4)	250 931 (8.8)	221 234 (8.1)
Diabetes	196 737 (3.5)	98 036 (3.4)	98 701 (3.6)
Dyslipidemia	976 236 (17.5)	507 758 (17.8)	468 478 (17.1)
Hypertension	999 936 (17.9)	526 726 (18.5)	473 210 (17.2)
Hyperuricemia	287 990 (5.2)	115 015 (4.0)	172 975 (6.3)

Abbreviations: BMI, body mass index (calculated as weight in kilograms divided by height in meters squared); BP, blood pressure; CKD, chronic kidney disease; eGFR, estimated glomerular filtration rate; HDL-C, high-density lipoprotein cholesterol; LDL-C, low-density lipoprotein cholesterol. To convert hemoglobin to g/L, multiply by 10; to convert cholesterol to mmol/L, multiply by 0.0259; to convert triglycerides to mmol/L, multiply by 0.0113; to convert glucose to mmol/L, multiply by 0.0555; to convert uric acid to mmol/L, multiply by 0.0595.

### Income Levels and Risk of Impaired Kidney Function

**Figure 1.**
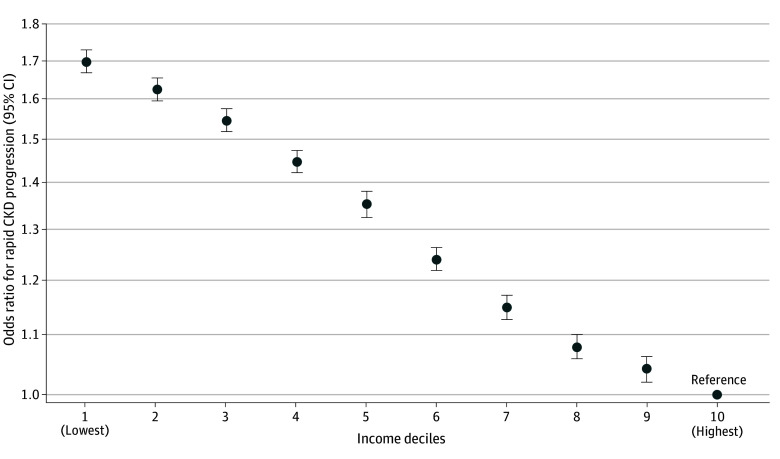
Association Between Individual Income Levels and Rapid Chronic Kidney Disease (CKD) Progression The y-axis shows the log scale of the odds ratio; circles are the point estimates; and error bars indicate the 95% CIs. The model was adjusted for age, sex, smoking, body mass index, waist circumference, hemoglobin, systolic blood pressure, low-density lipoprotein cholesterol, high-density lipoprotein cholesterol, triglycerides, blood glucose, uric acid, diabetes, hypertension, cardiovascular disease, cancer, dyslipidemia, hyperuricemia, and prefecture. The lowest income groups showed the largest odds ratio for rapid CKD progression, and the association was negative monotonic.

**Figure 2.**
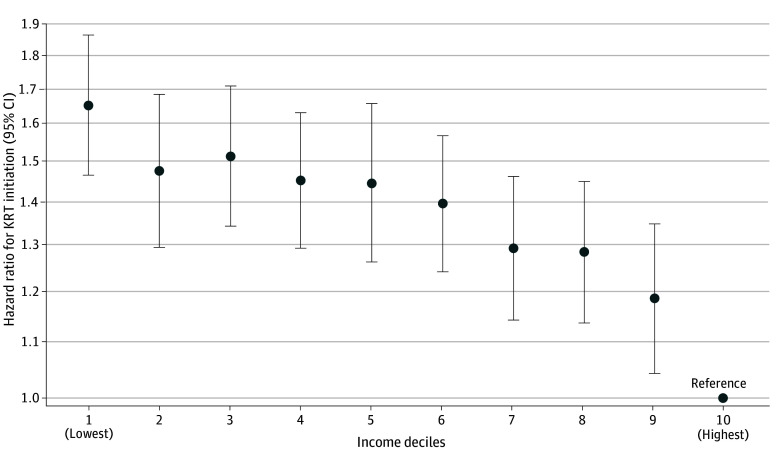
Association Between Individual Income Levels and Initiation of Kidney Replacement Therapy (KRT) The y-axis shows the log scale of the hazard ratio; circles are the point estimates; and error bars indicate the 95% CIs. The model was adjusted for age, sex, smoking, body mass index, waist circumference, hemoglobin, systolic blood pressure, low-density lipoprotein cholesterol, high-density lipoprotein cholesterol, triglycerides, blood glucose, uric acid, diabetes, hypertension, cardiovascular disease, cancer, dyslipidemia, hyperuricemia, and prefecture. The lowest income groups showed the largest hazard ratio for initiation of KRT, and the association was negative monotonic with a gradual slope.

During the study period, the mean (SD) eGFR measurement was 5.5 (1.8) times, with the mean (SD) interval between the first and last measurements being 4.7 (1.8) years. Rapid CKD progression was observed among 323 686 individuals (201 465 males [5.4%] and 122 221 females [6.5%]). Adjusting for potential confounders using a logistic regression model, the lowest income group showed the largest odds ratio (OR, 1.70; 95% CI, 1.67-1.73) for rapid CKD progression (Figure 1). During a median (IQR) follow-up of 6.3 (4.1-6.6) years, 5939 individuals (5256 males [0.14%] and 683 females [0.04%]) underwent KRT, and the lowest income group also showed the largest hazard ratio (HR, 1.65; 95% CI, 1.47-1.86) (Figure 2). Schoenfeld residuals provide no evidence of the violation of the proportional assumption (eTable 5 in Supplement 1). The annual eGFR decline volume was the largest in the lowest income group, with 1.02 (95% CI, 1.01-1.02) mL/min/1.73 m^2^/y, and the smallest in the highest income group, with 0.94 (95% CI, 0.93–0.95) mL/min/1.73 m^2^/y; a difference of 0.08 (95% CI, 0.07-0.09) using a linear regression model (eFigure 2 in Supplement 1). A negative monotonic association between income and the development of impaired kidney function was observed for all outcomes.

### Subgroup Analyses by Sex

**Figure 3.**
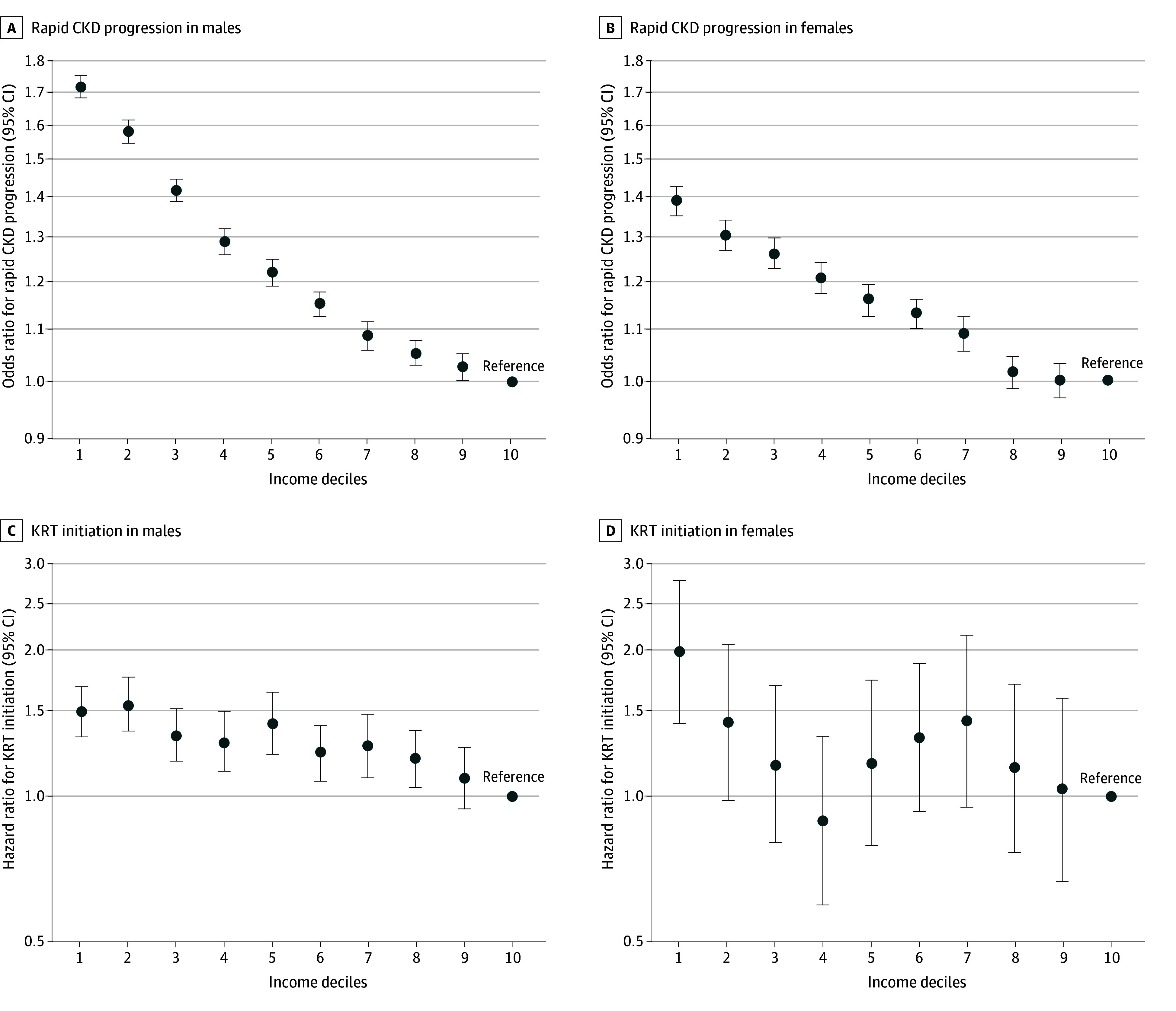
Subgroup Analysis for the Association Between Income and Impaired Kidney Function, by Sex A and B show adjusted odds ratio for rapid CKD progression by income levels; and C and D, adjusted hazard ratio for the initiation of KRT by income levels. The y-axis shows the log scale of the odds ratio (A, B) and the hazard ratio (C, D); circles are the point estimates; and error bars indicate the 95% CIs. An income decile of 1 indicates the lowest, and 10 indicates the highest. Volumes were adjusted for age, smoking, body mass index, waist circumference, hemoglobin, systolic blood pressure, low-density lipoprotein cholesterol, high-density lipoprotein cholesterol, triglycerides, blood glucose, uric acid, diabetes, hypertension, cardiovascular disease, cancer, dyslipidemia, hyperuricemia, and prefecture. Males showed a stronger association between individual income and impaired kidney function than females, while both subgroups showed a clear negative monotonic association for rapid CKD progression. Although an increased risk for initiation of KRT was observed in the first to second decile of income levels in both sexes, the trend was not clear, particularly for female individuals due to a small number of events. *P* for interaction was detected as <.001 for rapid CKD progression and 0.12 for initiation of KRT. CKD indicates chronic kidney disease; KRT, kidney replacement therapy.

Overall, income disparities were more prominent for males than females (Figure 3). The ORs for rapid CKD progression in the lowest income groups were 1.72 (95% CI, 1.68-1.75) among males and 1.39 (95% CI, 1.35-1.43) among females (*P* for interaction < .001) compared with the highest income group. The differences between income groups were larger for males than for females while both subgroups showed a clear negative monotonic association. The HRs for the initiation of KRT in the lowest income groups were 1.49 (95% CI, 1.33-1.68) among males and 1.98 (95% CI, 1.42-2.78) among females (*P* for interaction = .12), compared with the highest income group. Although we observed an increased risk in the first through second decile of income levels in both sexes, the trend was not clear, particularly for females because of the small number of events.

### Subgroup Analyses by Diabetes Status

**Figure 4.**
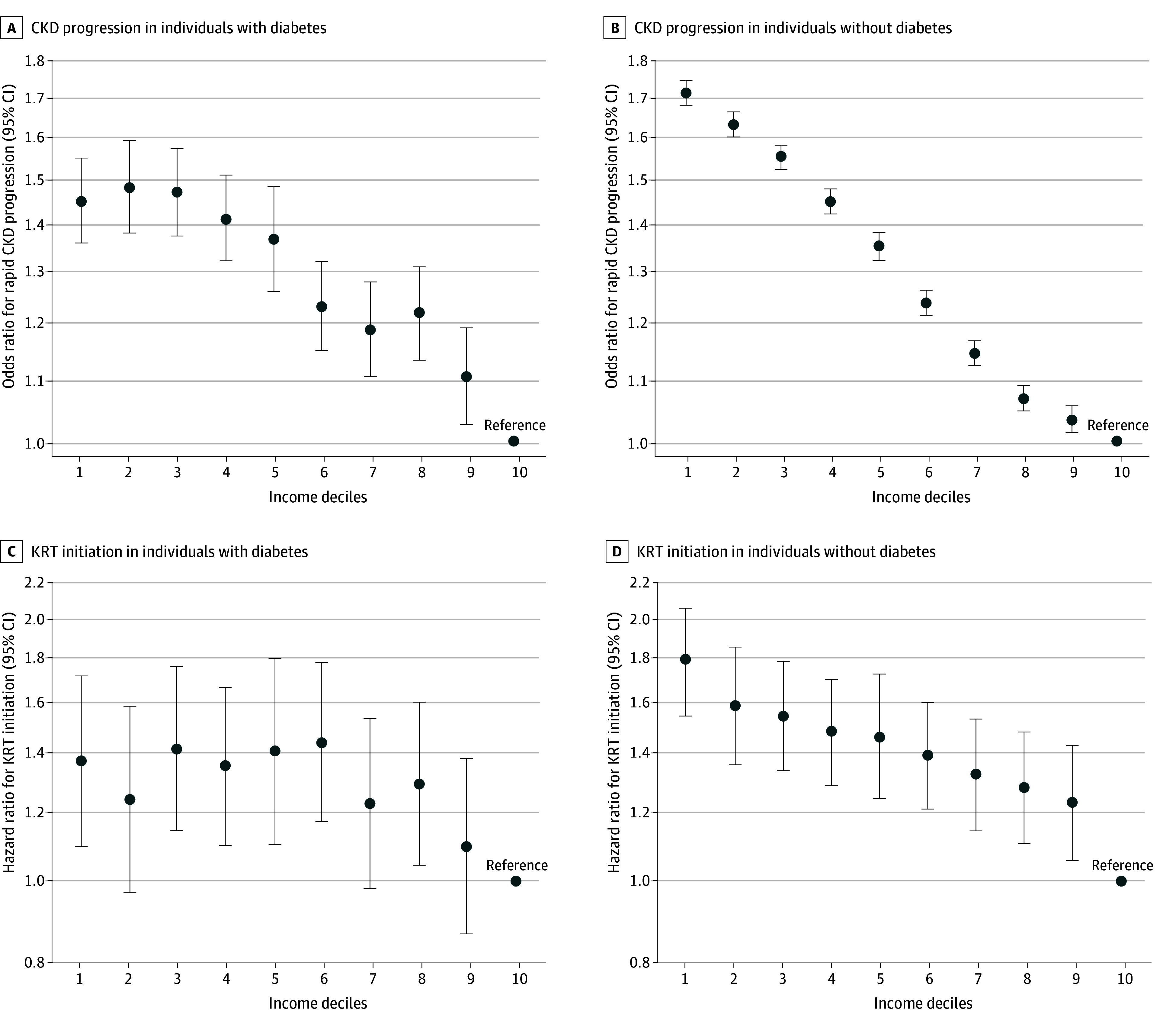
Subgroup Analysis for the Association Between Income and Impaired Kidney Function by Diabetes Status A and B show adjusted odds ratio for rapid CKD progression by income levels; and C and D, adjusted hazard ratio for the initiation of KRT by income levels. The y-axis shows the log scale of the odds ratio (A, B) and hazard ratio (C, D); circles are the point estimates; and error bars indicate the 95% CIs. An income decile of 1 indicates the lowest, and 10 indicates the highest. Volumes were adjusted for age, sex, smoking, body mass index, waist circumference, hemoglobin, systolic blood pressure, low-density lipoprotein cholesterol, high-density lipoprotein cholesterol, triglycerides, blood glucose, uric acid, hypertension, cardiovascular disease, cancer, dyslipidemia, hyperuricemia, and prefecture. CKD indicates chronic kidney disease; KRT, kidney replacement therapy. Individuals without diabetes showed a stronger association between individual income levels and impaired kidney function than did individuals with diabetes. Although a negative monotonic association was observed among individuals without diabetes, the first to fifth deciles among individuals with diabetes showed a similar risk for impaired kidney function, with a more gradual slope across income levels. *P* for interaction was detected as <.001 for rapid CKD progression and .06 for initiation of KRT. CKD indicates chronic kidney disease; KRT, kidney replacement therapy.

The income disparities were greater among individuals without diabetes than those with diabetes (Figure 4). As for rapid CKD progression, the OR for the lowest income group was 1.72 (95% CI, 1.68-1.75) among individuals without diabetes and 1.45 (95% CI, 1.36-1.55) for those with diabetes (*P* for interaction < .001) compared with those in the highest income group. The HR for the initiation of KRT in the lowest income groups (vs the highest income group) was 1.78 (95% CI, 1.54-2.05) among individuals without diabetes and 1.37 (95% CI, 1.09-1.72) among individuals with (*P* for interaction = .06). Although a negative monotonic association was observed among individuals without diabetes, the first to fifth deciles among individuals with diabetes showed a similar risk increasing of impaired kidney function, with a more gradual slope across income levels.

### Subgroup Analyses by Baseline CKD Stage

For all outcomes, the association of income and impaired kidney function differed by baseline CKD stage (eFigure 3 in Supplement 1). The OR for rapid CKD progression for the lowest income group was 1.70 (95% CI, 1.67-1.73) among individuals with early CKD (stage 1-2) and 1.56 (95% CI, 1.43-1.70) for those with advanced CKD (stage 3-5) (*P* for interaction = .06), compared with those in the highest income group. The HR for the initiation of KRT in the lowest income groups (vs the highest income group) was 1.30 (95% CI, 0.92-1.84) among individuals with early CKD (stages 1-2) and 1.50 (95% CI, 1.32–1.71) among individuals with advanced CKD (stages 3-5) (*P* for interaction = .45). For rapid CKD progression, we observed a negative monotonic increase in risks by income levels regardless of baseline CKD stage. For initiation of KRT, although no significant differences were found among the income groups for individuals at early CKD stage at the baseline, probably because of the small prevalence of KRT, a modest negative monotonic association was observed for individuals at advanced CKD stage at the baseline.

### Sensitivity Analysis

When outcome variables were adjusted for urinary protein levels and baseline eGFR values, we found qualitatively consistent results while the estimates decreased toward the null especially for the initiation of KRT (eFigure 4 and eFigure 5 in Supplement 1). The results were also consistent when we used the inverse probability weighting approach (eFigure 6 in Supplement 1) and when we used average income over the study period rather than income in the fiscal year 2015 (eFigure 7 in Supplement 1).

### Additional Analysis

When we stratified by urinary protein levels, we found a clear negative monotonic increase in risk among those without proteinuria while the association was less clear among those with proteinuria for rapid CKD progression (eFigure 8 in Supplement 1). The Slope Index of Inequality was 349.5 cases per 10 000 persons for rapid CKD progression, and 95.2 cases per million person-years for KRT initiation, respectively (eTable 6 in Supplement 1). When we simulated the population effects of the increased CKD disparities across income levels, the adjusted absolute risk difference for rapid CKD progression was 301.2 (95% CI, 291.9-310.5) cases per 10 000 persons between the lowest income group and the highest income group (eTable 7 in Supplement 1). The population-attributable risk (PAR) was 22.6% (73 112 cases) in rapid CKD progression and 26.4% (1568 cases) in KRT initiation, respectively, assuming the highest income group (top 10th percentile) as an unexposed population (eTable 8 in Supplement 1). The PAR was 15.5% (50 225 cases) in rapid CKD progression and 9.8% (581 cases) in KRT initiation, respectively, assuming income greater than the median (top 50th percentile) as an unexposed population.

## Discussion

Using a nationwide retrospective cohort in Japan, we found that lower income levels were associated with faster CKD progression and a higher risk of initiation of KRT. The differences in rapid CKD progression and initiation of KRT across income levels were large—the lowest income group’s risks were more than 60% higher compared with the risks of the highest income group, even in a country where universal health coverage has been mostly achieved. A negative monotonic association was more pronounced among males and individuals without diabetes. The association of income with rapid CKD progression was observed among both individuals at early CKD stage and those at advanced CKD stage, indicating the presence of income inequalities for both development of CKD and progression of existing CKD.

Our findings are consistent with findings from other high-income countries, including the US, Sweden, and South Korea,^10,11,12,13,16,17^ which suggests that income poverty is a risk factor for CKD progression. Moreover, our findings indicate that this income disparity is not only associated with financial barriers to medical care because Japan has achieved universal health coverage since 1961 and has few financial barriers to medical services—medical expenses are covered by the government and/or insurers, and there is a legal limit on the maximum medical expenses that insured individuals would have to pay per month. In addition, annual health examinations for all insured individuals are mandated by law, ensuring opportunities for secondary prevention. Even under the extensive health care system in Japan, which differs from other countries, it is noteworthy that large income disparities persist in terms of impaired kidney function. This finding provides crucial information for considering future health care policies. Low income may underpin scarcity of material goods, lower health literacy, higher psychosocial strain,^34^ and physiological burdens, and stress-related behavioral risks, which during a lifetime could be associated with the severe consequences of CKD progression.^35,36,37^ Given that CKD is a generally progressive but irreversible condition with implicit and substantial health care costs, more active support—beyond financial assistance to ensure access to medical care and secondary prevention services—may be needed to prevent CKD and its progression.

In our subgroup analyses, we found income disparity in that impaired kidney function was larger in the nondiabetes subgroup, a finding consistent with a report from South Korea.^17^ These findings can be partially explained by that diabetes is a strong prognostic factor for CKD progression^38^; individuals with diabetes already have a higher risk of developing CKD and initiating KRT than individuals without diabetes have at the baseline, and thus the role of income levels could be relatively small. Another potential explanation is that individuals with underdiagnosed diabetes were miscategorized into the nondiabetes group because our categories were based on *ICD-10* codes reported in the insurance claims. Assuming worse health care access for more disadvantaged individuals could obscure the existing income inequalities in the group with diabetes. We also found a greater income disparity in the development of impaired kidney function among males than among females, which is different from the previously mentioned finding of no clear sex differences between individual income levels and CKD incidence.^17^ Although the underlying mechanisms are not clear, this observed heterogeneity may be partially explained by the sample selection (restricting the sample to regular employees with long working hours contributed to a high prevalence of male individuals), measurement of individual-level income levels (that do not reflect spousal or family income levels), and biological and behavioral sex differences (males have a higher risk of CKD progression than do females).^39^ From the subgroup analyses by baseline CKD stage, income disparities were observed both in early-stage CKD and in advanced-stage CKD. This finding is consistent with those of previous studies and implies the importance of considering socioeconomic status to prevent the development and progression of CKD.^20^

The findings of this study have several policy implications. First, the study supports the World Health Organization’s recommendation to address the social determinants of health.^40^ Second, it draws attention to the fact that economic guarantees for medical care and secondary prevention opportunities may be insufficient for preventing the onset and progression of CKD. Although the strong association we observed between low income and the development of impaired kidney function does not necessarily imply causality, further society-wide interventions to reduce poverty, poverty-related social stress, and social-behavioral risks need to be considered. The income redistribution rate in Japan is relatively lower than that of other high-resource countries^41^; thus, there may be scope for further equalization. In Japan, the government has launched several strategies to address CKD since 2008,^42^ reducing the age-adjusted number of patients commenced on dialysis.^43^ However, the findings of this study suggest there is a need to focus on socioeconomically disadvantaged populations.

### Limitations

Despite the strength of this study—large sample size and nearly complete availability of eGFR and urinary protein data among individuals with health examination data—several limitations should be noted. First, given that only individuals who had undergone health examinations were included in the study sample, the findings may be distorted by self-selection bias. Individuals with low socioeconomic status conventionally have lower rates of health screening.^44^ In addition, given the possibility that these individuals were more likely to discontinue participation, attrition bias may have affected the findings. Second, we were unable to exclude the possibility of measurement errors in income levels and outcomes because income level was assessed only at baseline and did not cover household income, and kidney function was measured only once a year. Future studies are needed to account for the time-varying nature of income levels. Third, although we adjusted for an extensive set of covariates, including comorbidities from medical claim data and biological data from annual health examinations, the severity of comorbidities and educational status were not reported in these data, raising concerns regarding unmeasured and residual confounding. Lastly, because we used a database of the working-age population, our findings are not necessarily generalizable to other populations.

## Conclusions

The findings of this retrospective cohort study suggest there was a substantial association between low-income levels and the development of impaired kidney function, with rapid CKD progression and initiation of KRT. These findings highlight the importance of society-wide interventions aimed at reducing the social and behavioral risks to individuals in the low-income group in addition to financial guarantee of access to medical care and disease prevention.

## Notes

Supplement 1. eMethod 1. Database of the Japan Health Insurance Association eMethod 2. Details in eGFR calculation eMethod 3. Sensitivity analyses eMethod 4. Additional analyses eFigure 1. Flow of study sample selection eFigure 2. The estimated average estimated glomerular filtration rate (eGFR) decline volume per year by individual income levels eFigure 3. Subgroup analysis for the association between income and impaired kidney function by baseline CKD stage eFigure 4. Sensitivity analysis additionally adjusting for urinary protein levels eFigure 5. Sensitivity analysis additionally adjusting for baseline estimated glomerular filtration rate (eGFR) eFigure 6. Sensitivity analysis using inverse probability weighting approach for missing data of covariates and outliers in laboratory data eFigure 7. Sensitivity analysis according to average annual income during the study period eFigure 8. Subgroup analysis for the association between income and impaired kidney function by urinary protein levels eTable 1. Demographic characteristics of the study population by individual income levels eTable 2. Demographic characteristics of the study population by individual income levels among males eTable 3. Demographic characteristics of the study population by individual income levels among females eTable 4. Demographic characteristics of the study population taking health examinations and not taking health examinations eTable 5. Results of the proportional hazard tests based on Schoenfeld residuals between income deciles eTable 6. Slope and relative index of inequalities for impaired kidney function by income levels eTable 7. Absolute risk difference for rapid CKD progression between the 1st to 9th decile and the 10th decile eTable 8. Population-attributable risks of rapid chronic kidney disease (CKD) progression and kidney replacement therapy (KRT) initiation by setting income levels at top 10th and 50th percentiles

Supplement 2. Data Sharing Statement
