# Supplementary material for: Income Level and Impaired Kidney Function Among Working Adults in Japan
Source: JAMA Health Forum. 2024 Mar 1;5(3):e235445. doi: 10.1001/jamahealthforum.2023.5445 (PMC10907921; doi:10.1001/jamahealthforum.2023.5445)
Supplement: Supplement 1. — eMethod 1. Database of the Japan Health Insurance Association eMethod 2. Details in eGFR calculation eMethod 3. Sensitivity analyses eMethod 4. Additional analyses eFigure 1. Flow of study sample selection eFigure 2. The estimated average estimated glomerular filtration rate (eGFR) decline volume per year by individual income levels eFigure 3. Subgroup analysis for the association between income and impaired kidney function by baseline CKD stage eFigure 4. Sensitivity analysis additionally adjusting for urinary protein levels eFigure 5. Sensitivity analysis additionally adjusting for baseline estimated glomerular filtration rate (eGFR) eFigure 6. Sensitivity analysis using inverse probability weighting approach for missing data of covariates and outliers in laboratory data eFigure 7. Sensitivity analysis according to average annual income during the study period eFigure 8. Subgroup analysis for the association between income and impaired kidney function by urinary protein levels eTable 1. Demographic characteristics of the study population by individual income levels eTable 2. Demographic characteristics of the study population by individual income levels among males eTable 3. Demographic characteristics of the study population by individual income levels among females eTable 4. Demographic characteristics of the study population taking health examinations and not taking health examinations eTable 5. Results of the proportional hazard tests based on Schoenfeld residuals between income deciles eTable 6. Slope and relative index of inequalities for impaired kidney function by income levels eTable 7. Absolute risk difference for rapid CKD progression between the 1st to 9th decile and the 10th decile eTable 8. Population-attributable risks of rapid chronic kidney disease (CKD) progression and kidney replacement therapy (KRT) initiation by setting income levels at top 10th and 50th percentiles [file jamahealthforum-e235445-s001.pdf]

Supplemental Materials

Ishimura N, Inoue K, Maruyama S, Nakamura S, Kondo N. Income Level and Impaired Kidney Function Among Working Adults in Japan. *JAMA Health Forum*. Published online March 1, 2024. doi:10.1001/jamahealthforum.2023.5445

eMethod 1. Database of the Japan Health Insurance Association

eMethod 2. Details in eGFR calculation

eMethod 3. Sensitivity analyses

eMethod 4. Additional analyses

eFigure 1. Flow of study sample selection

eFigure 2. The estimated average estimated glomerular filtration rate (eGFR) decline volume per year by individual income levels

eFigure 3. Subgroup analysis for the association between income and impaired kidney function by baseline CKD stage

eFigure 4. Sensitivity analysis additionally adjusting for urinary protein levels

eFigure 5. Sensitivity analysis additionally adjusting for baseline estimated glomerular filtration rate (eGFR)

eFigure 6. Sensitivity analysis using inverse probability weighting approach for missing data of covariates and outliers in laboratory data

eFigure 7. Sensitivity analysis according to average annual income during the study period

eFigure 8. Subgroup analysis for the association between income and impaired kidney function by urinary protein levels

eTable 1. Demographic characteristics of the study population by individual income levels

eTable 2. Demographic characteristics of the study population by individual income levels among males

eTable 3. Demographic characteristics of the study population by individual income levels among females

eTable 4. Demographic characteristics of the study population taking health examinations and not taking health examinations

eTable 5. Results of the proportional hazard tests based on Schoenfeld residuals between income deciles

© 2024 Ishimura N et al. *JAMA Health Forum*

eTable 6. Slope and relative index of inequalities for impaired kidney function by income levels

eTable 7. Absolute risk difference for rapid CKD progression between the 1st to 9th decile and the 10th decile

eTable 8. Population attributable risks of rapid chronic kidney disease (CKD) progression and kidney replacement therapy (KRT) initiation by setting income levels at top 10th and 50th percentiles

This supplemental material has been provided by the authors to give readers additional information about their work.

© 2024 Ishimura N et al. *JAMA Health Forum*

eMethod 1. Database of the Japan Health Insurance Association

The Japan Health Insurance Association (JHIA) is the largest public medical insurer in Japan, covering approximately 40% (30 million) of the working-age population. To qualify for the JHIA coverage, individuals need to work a certain number of hours at these companies. They lose this entitlement if they leave their job, for example, to start a new job at a large company or due to health issues such as illness. Members of the JHIA are eligible to receive an annual health check-up, as mandated to be provided by insurers by law. The participation rate for health check-ups among JHIA-insured individuals aged 40 to 74 years (target ages for specific health check-ups to prevent lifestyle diseases mandated by law), after excluding those who joined or withdrew during the year and those who had difficulty taking the check-ups due to long-term hospitalization or other reasons, was 53.8% (2015).[1]

References:

1. Ministry of Health, Labour and Welfare. Implementation Status of Specific Health Check-ups and Specific Health Guidance in Fiscal Year 2015. Updated July 31, 2017. https://www.mhlw.go.jp/file/04-Houdouhappyou-12401000-Hokenkyoku-Soumuka/0000173093.pdf (Japanese). Accessed May 24, 2023.

© 2024 Ishimura N et al. *JAMA Health Forum*

eMethod 2. Details in eGFR calculation

The eGFR was calculated using a creatinine formula modified for Japanese individuals: eGFR = $194 \times \text{serum creatinine}^{-1.094} \times \text{age}^{-0.287} \times \alpha$ ($\alpha = 0.739$ for women, and $\alpha = 1$ for men).[1] We adopted a single eGFR measurement from the first health check-up of each individual in each fiscal year. The annual decline in eGFR (mL/min/1.73m^2) was calculated based on the interval between the baseline check-up in the fiscal year 2015 and the check-up in the latest fiscal year during the fiscal years 2016 to 2021 for each individual. We distinguished continuous dialysis from temporary dialysis according to whether individuals underwent dialysis for at least two consecutive months. We right-censored individuals when they died or withdrew from insurance, due to job termination or other reasons.

References:

1. Matsuo S, Imai E, Horio M, et al. Revised equations for estimated GFR from serum creatinine in Japan. *Am J Kidney Dis*. 2009;53(6):982-992.

© 2024 Ishimura N et al. JAMA Health Forum

eMethod 3. Sensitivity analyses

We conducted the following three sensitivity analyses. First, to account for residual confounding we adjusted for urinary protein levels (categorical 5-level variables based on random spot urinalysis; -, ±, 1+, 2+, 3+) and baseline eGFR values in the regression models. Second, to address the selection bias by excluding individuals due to missing covariate data and laboratory outliers, we conducted an analysis using the Inverse Probability Weighting (IPW) approach.[1] Third, to account for changes in income levels over the study period, we reanalyzed the data using the average income for the study period instead of the individual incomes in the fiscal year 2015 as an exposure.

References:

1. Seaman SR, White IR. Review of inverse probability weighting for dealing with missing data. *Stat Methods Med Res*. 2013;22(3):278-295.

© 2024 Ishimura N et al. *JAMA Health Forum*

eMethod 4. Additional analyses

We also conducted three additional analyses. First, to assess the heterogeneity by the urinary protein level, we conducted the subgroup analyses by baseline urinary protein levels: (-), (±) vs. (1+) or above. Second, to assess the degree of inequalities more formally, we calculated the Slope Index of Inequality (SII),[1] Relative Index of Inequality (RII),[2] and Kunst Mackenbach Relative Index (KMI).[3] For rapid CKD progression and KRT initiation, we calculated these indices using the prevalence (per 10,000 persons) and incidence (per million person-years), respectively. Third, to simulate the population impacts of the increased CKD disparities across income levels, we calculated adjusted absolute risk difference for rapid CKD progression and population attributable risk (PAR) for rapid CKD progression and KRT initiation, assuming the highest income group (top 10th percentile) or those above the median income (top 50th percentile) as an unexposed population.

References:

1. Preston SH, Haines MR, Pamuk E. *Effects of industrialization and urbanization on mortality in developed countries*. Department of Economics, Wayne State University; 1981.
2. Pamuk ER. Social-class inequality in infant mortality in England and Wales from 1921 to 1980. European Journal of Population/Revue Européenne de Démographie. 1988:1-21.
3. Kunst AE, Mackenbach JP. *Measuring socio-economic inequalities in health. Copenhagen, Denmark.* World Health Organization, Regional Office for Europe; 1995.

© 2024 Ishimura N et al. *JAMA Health Forum*

eFigure 1. Flow of study sample selection

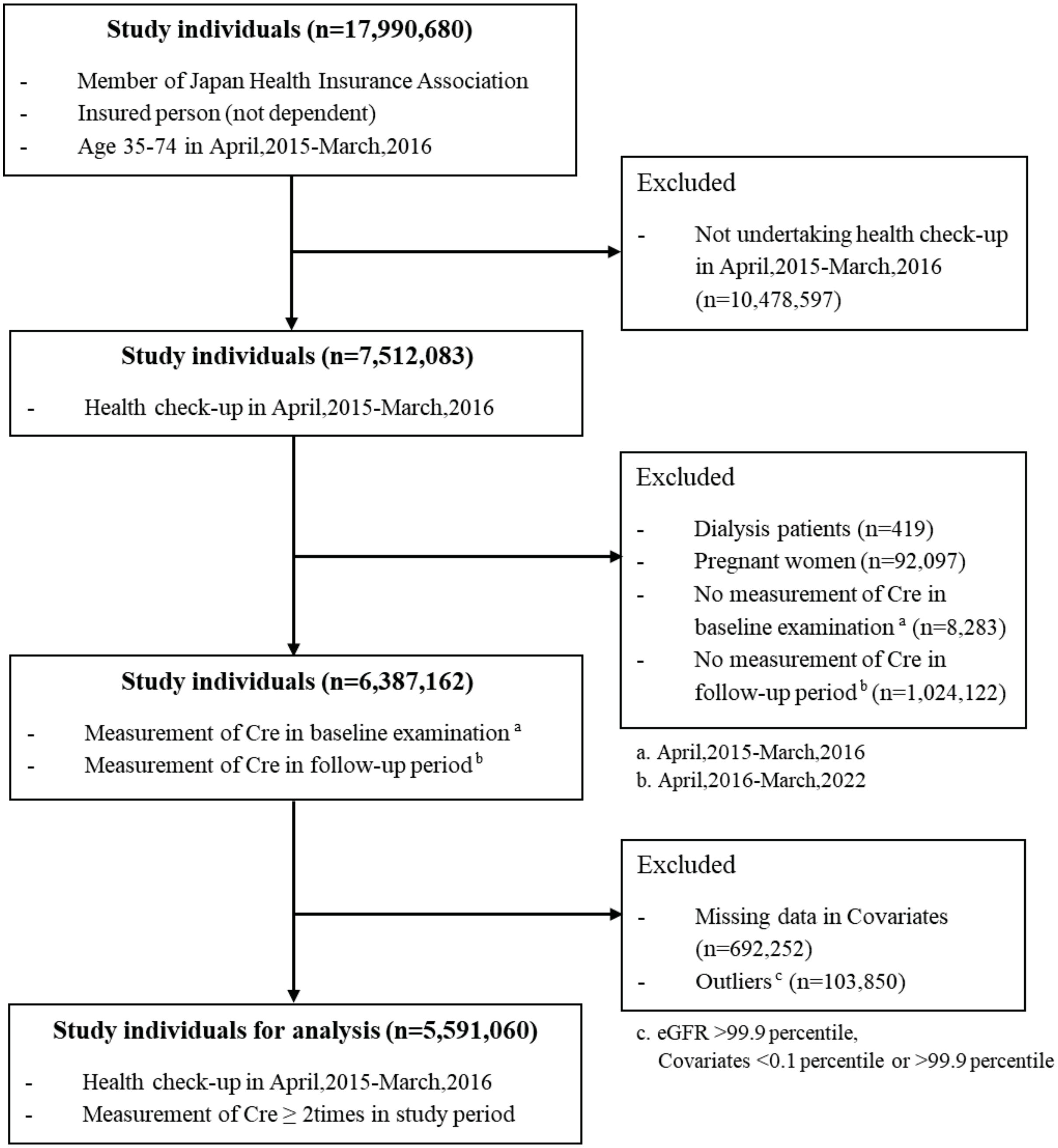

Cre=Creatinine, eGFR=estimated glomerular filtration rate

© 2024 Ishimura N et al. *JAMA Health Forum*

eFigure 2. The estimated average estimated glomerular filtration rate (eGFR) decline volume per year by individual income levels

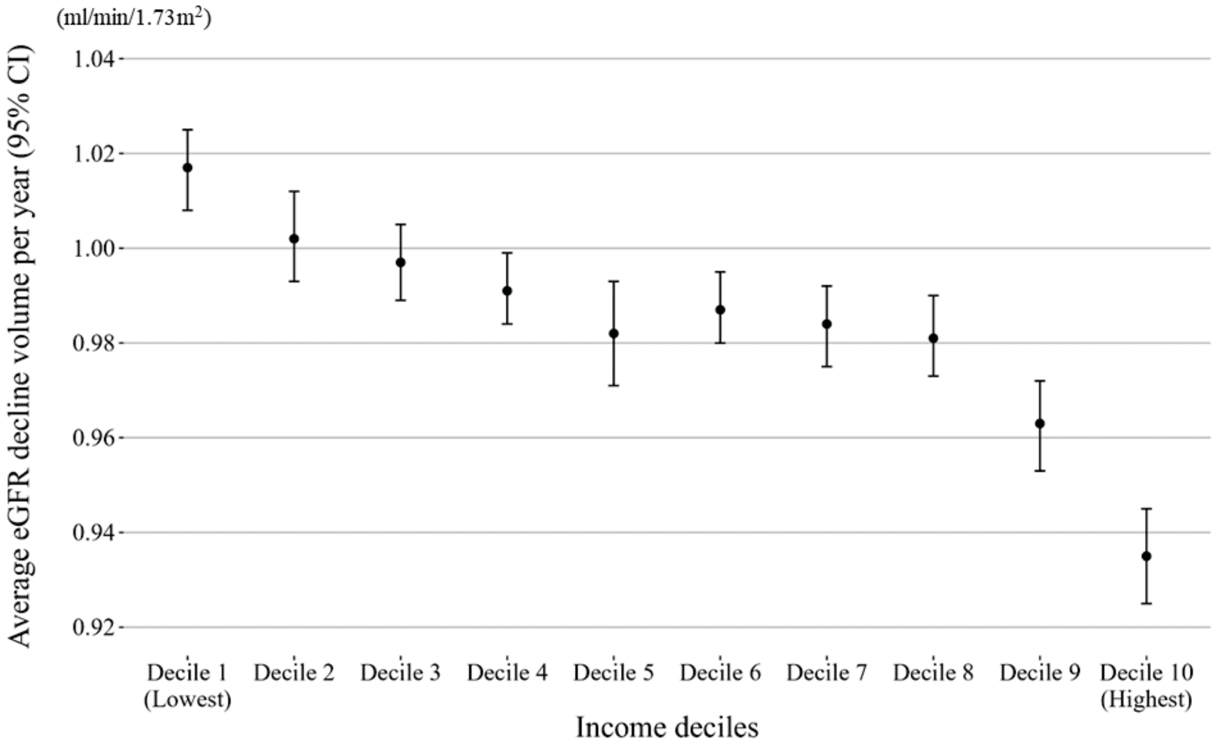

The model was adjusted for age, sex, smoking, BMI, waist circumference, hemoglobin, systolic blood pressure, LDL-cholesterol, HDL-cholesterol, triglyceride, blood glucose, uric acid, diabetes, hypertension, cardiovascular disease, cancer, dyslipidemia, hyperuricemia, and prefecture. The lowest income groups showed the largest decline in eGFR. eGFR=estimated glomerular filtration rate, BMI=body mass index, LDL=low-density lipoprotein and HDL=high-density lipoprotein

© 2024 Ishimura N et al. JAMA Health Forum

eFigure 3. Subgroup analysis for the association between income and impaired kidney function by baseline CKD stage

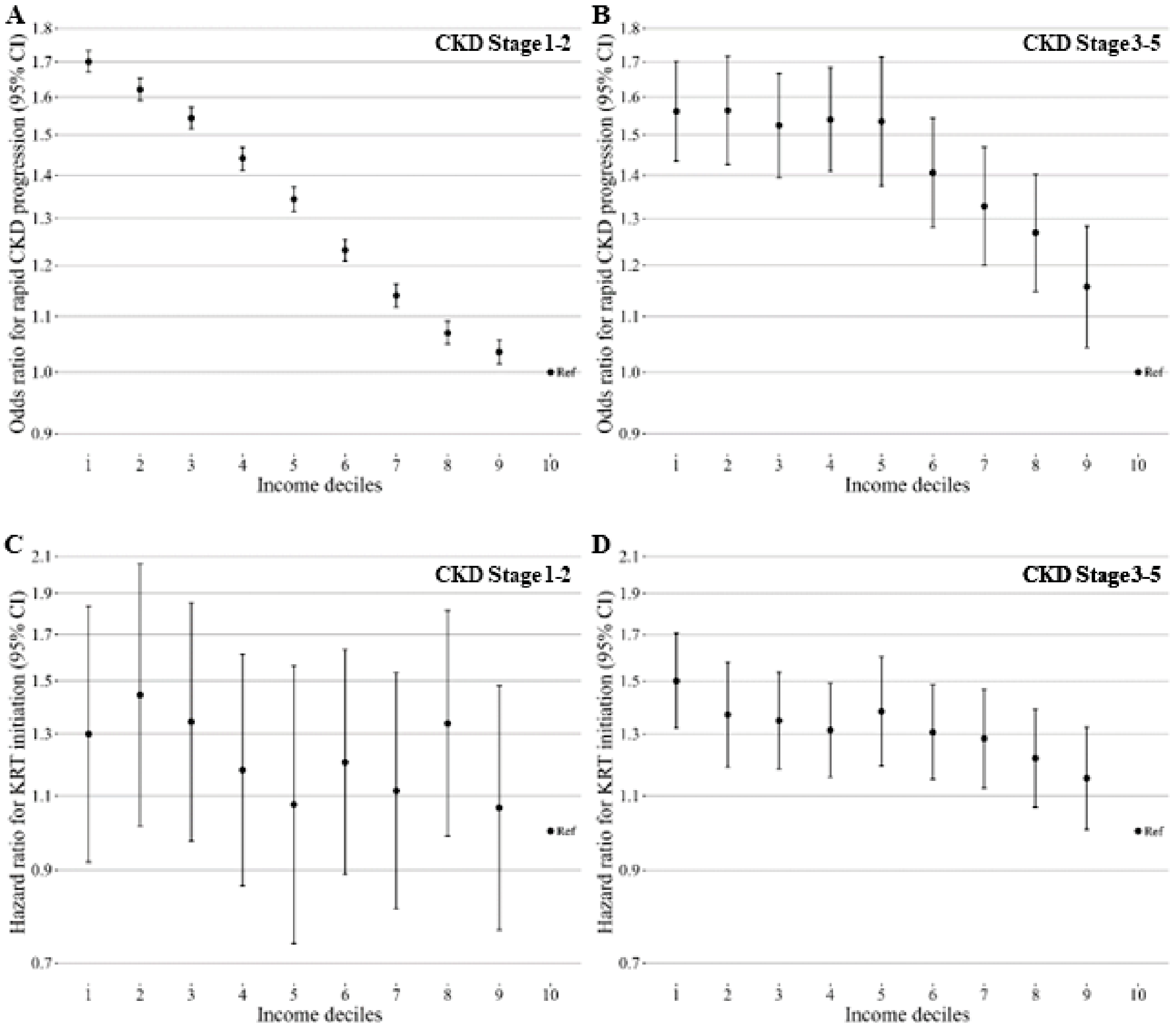

(A,B) Adjusted odds ratio for rapid CKD progression by income levels (C,D) Adjusted hazard ratio for the initiation of kidney replacement therapy (KRT) by income levels. Y-axis shows the log scale of the odds ratio (A, B) and the hazard ratio (C, D).

The volumes were adjusted for age, sex, smoking, BMI, waist circumference, hemoglobin, systolic blood pressure, LDL-cholesterol, HDL-cholesterol, triglyceride, blood glucose, uric acid, hypertension, cardiovascular disease, cancer, dyslipidemia, hyperuricemia, and prefecture. We found an association between income levels and both development of CKD and progression of existing CKD. The trend of association was different for each outcome by the baseline CKD stage. P-for-interaction was detected as .06 for rapid CKD progression and 0.45 for initiation of KRT.

eGFR= estimated glomerular filtration rate, CKD=chronic kidney disease, KRT=kidney replacement therapy, BMI=body mass index, LDL=low-density lipoprotein and HDL=high-density lipoprotein

© 2024 Ishimura N et al. *JAMA Health Forum*

eFigure 4. Sensitivity analysis additionally adjusting for urinary protein levels

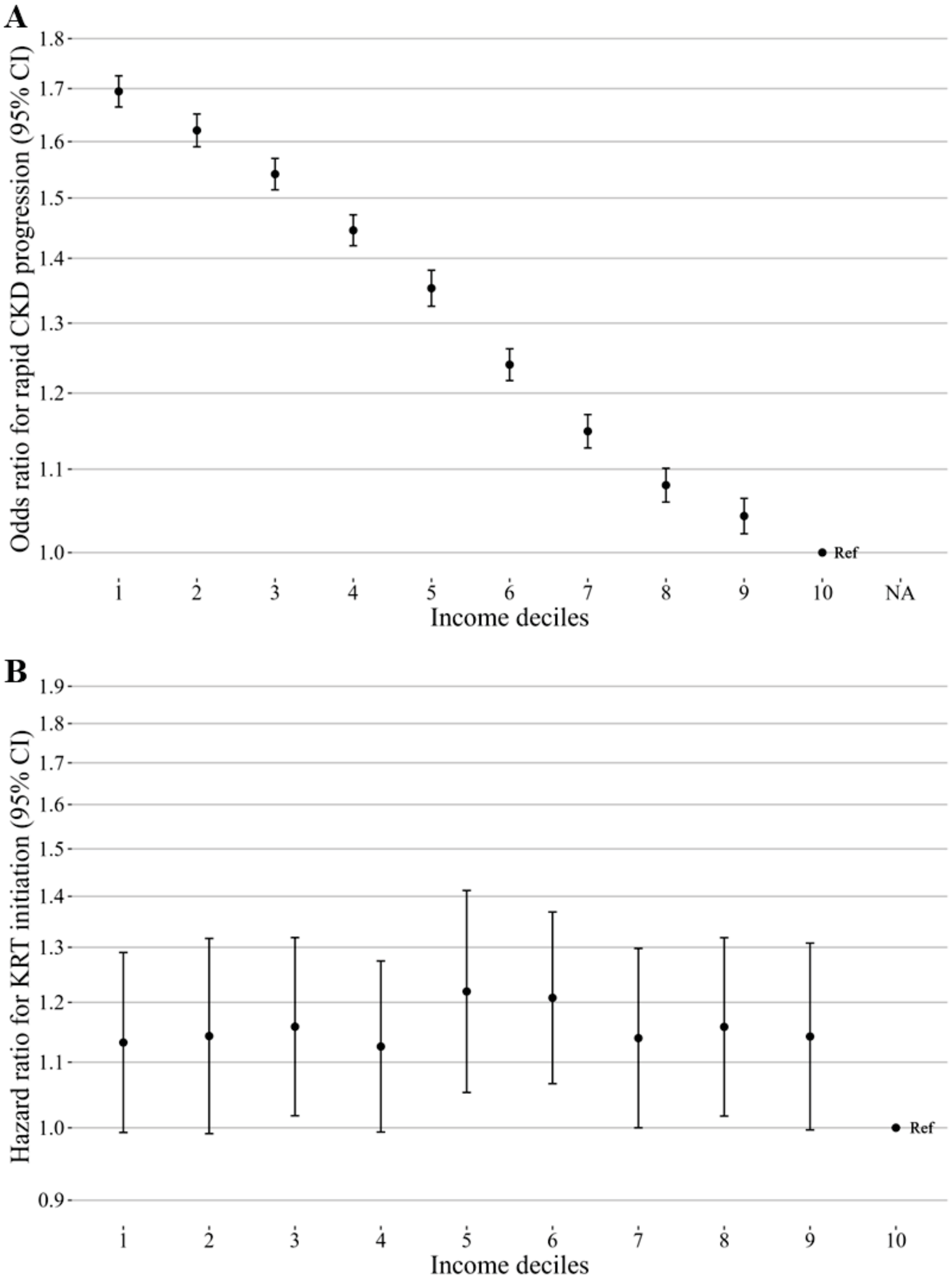

(A) Adjusted odds ratio for rapid CKD progression by income levels (B) Adjusted hazard ratio for initiation of kidney replacement therapy (KRT) by income levels. Y-axis shows the log scale of the odds ratio (A) and the hazard ratio (B).

The sample size was reduced to 5,566,791 due to the missing data of urinary protein, rapid CKD progression was observed in 322,222 cases (5.8 %) and KRT initiation was observed in 4,958 cases (0.1 %).

eGFR= estimated glomerular filtration rate, CKD=chronic kidney disease and KRT=kidney replacement therapy

© 2024 Ishimura N et al. *JAMA Health Forum*

eFigure 5. Sensitivity analysis additionally adjusting for baseline eGFR

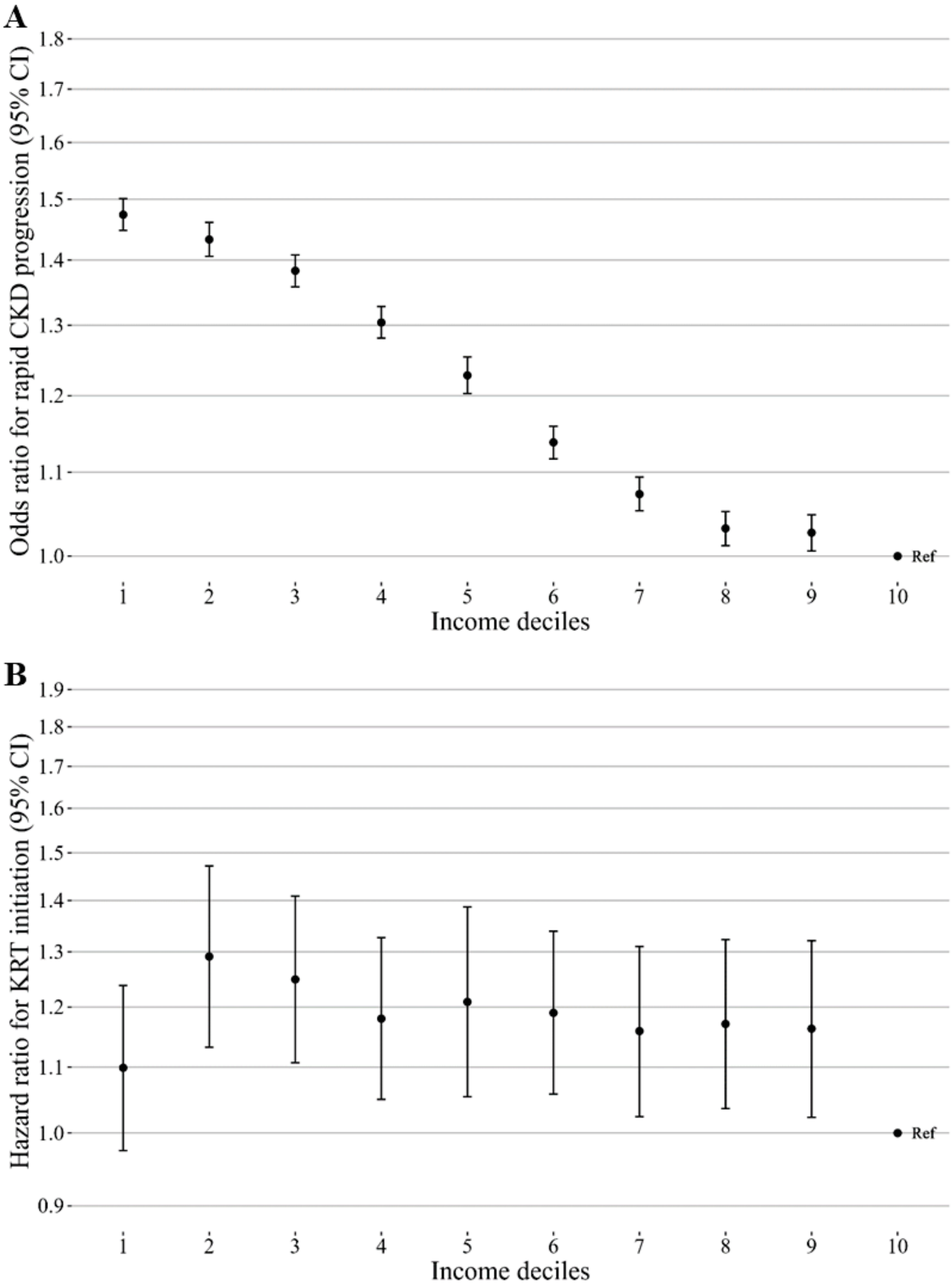

(A) Adjusted odds ratio for rapid CKD progression by income levels (B) Adjusted hazard ratio for initiation of kidney replacement therapy (KRT) by income levels. Y-axis shows the log scale of the odds ratio (A) and the hazard ratio (B).

eGFR= estimated glomerular filtration rate, CKD=chronic kidney disease and KRT=kidney replacement therapy

© 2024 Ishimura N et al. JAMA Health Forum

eFigure 6. Sensitivity analysis using inverse probability weighting approach for missing data of covariates and outliers in laboratory data

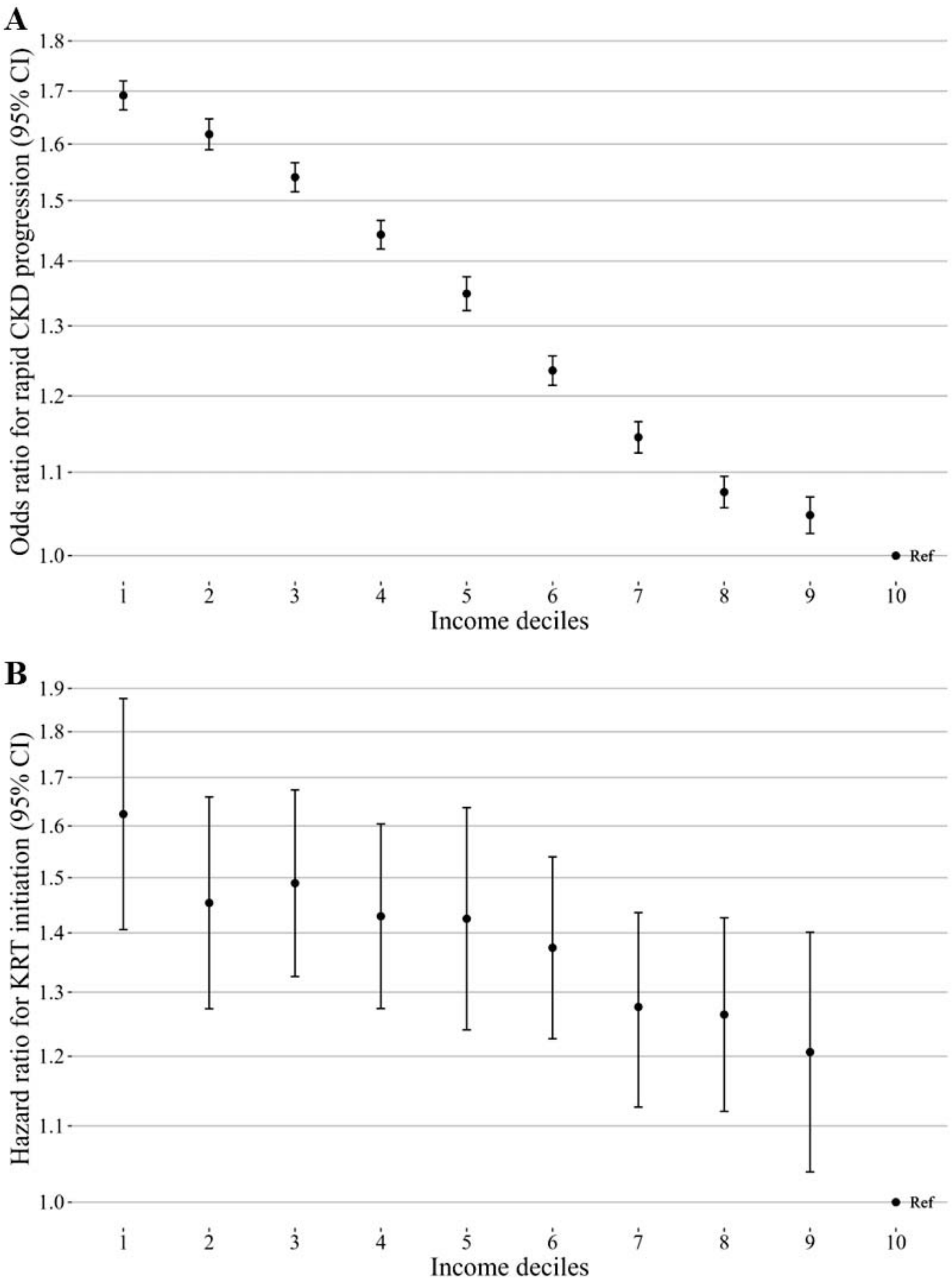

(A) Adjusted odds ratio for rapid CKD progression by income levels (B) Adjusted hazard ratio for the initiation of kidney replacement therapy (KRT) by income levels. Y-axis shows the log scale of the odds ratio (A) and the hazard ratio (B).

eGFR= estimated glomerular filtration rate, CKD=chronic kidney disease, KRT=kidney replacement therapy

© 2024 Ishimura N et al. *JAMA Health Forum*

eFigure 7. Sensitivity analysis according to average annual income during the study period

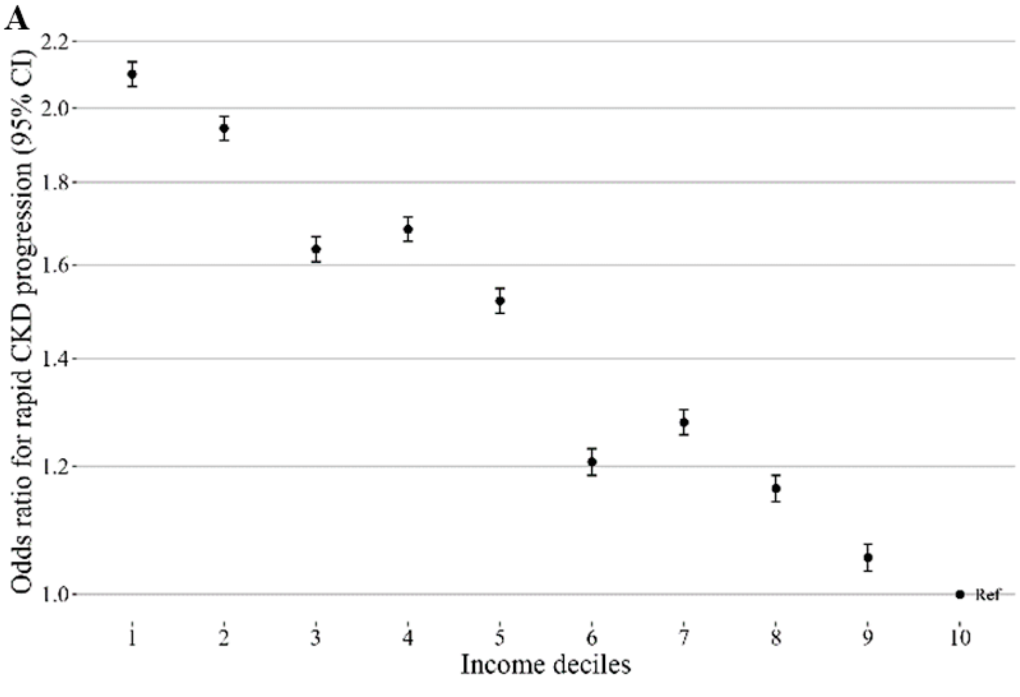

(A) Adjusted odds ratio for rapid CKD progression by income levels (B) Adjusted hazard ratio for the initiation of kidney replacement therapy (KRT) by income levels. Y-axis shows the log scale of the odds ratio (A) and the hazard ratio (B).

eGFR= estimated glomerular filtration rate, CKD=chronic kidney disease, KRT=kidney replacement therapy

© 2024 Ishimura N et al. *JAMA Health Forum*

eFigure 8. Subgroup analysis for the association between income and impaired kidney function by urinary protein levels

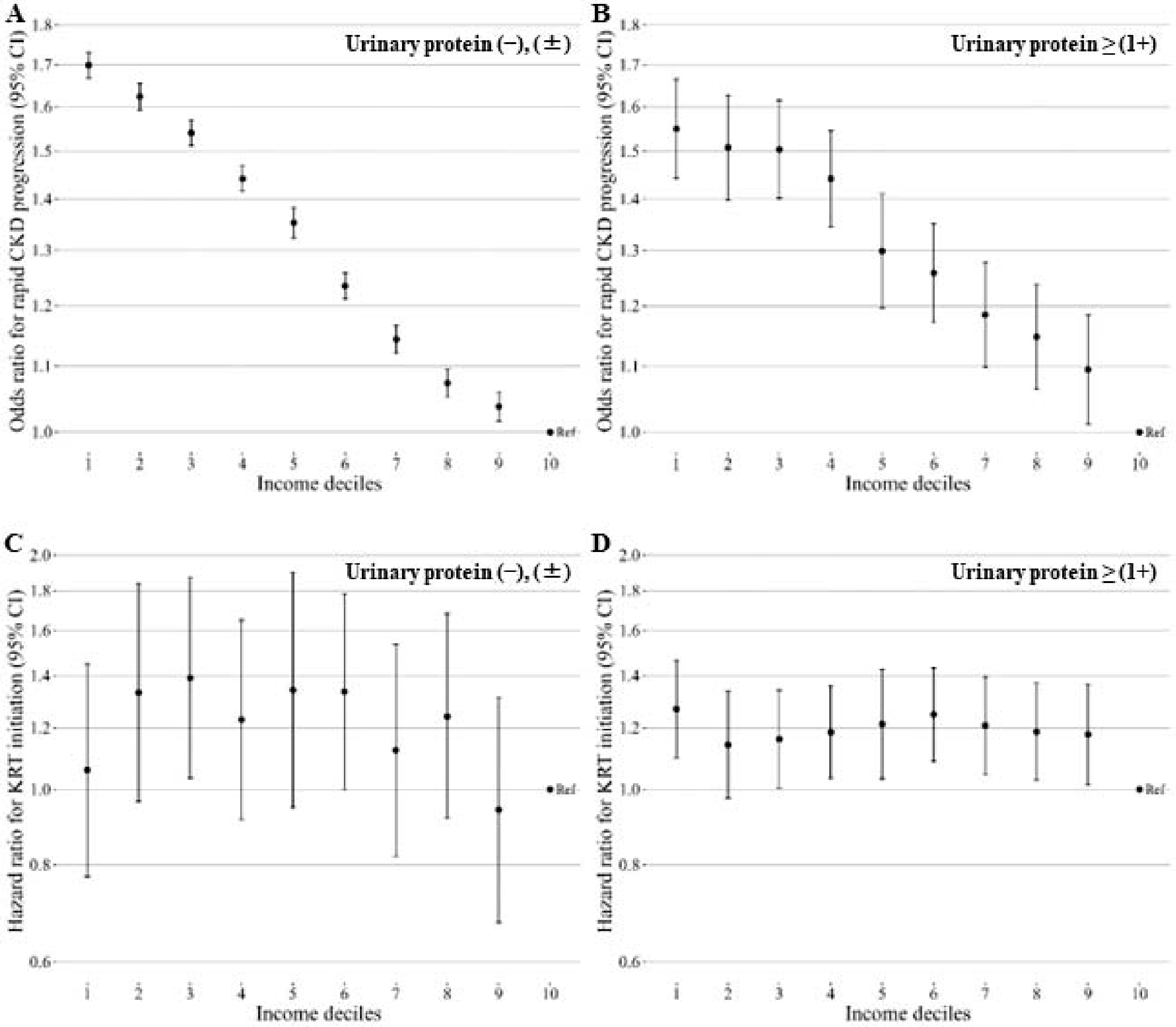

(A, B) Adjusted odds ratio for rapid CKD progression by income levels. (C,D) Adjusted hazard ratio for the initiation of kidney replacement therapy (KRT) by income levels. Y-axis shows the log scale of the odds ratio (A, B) and the hazard ratio (C, D).

The volumes were adjusted for age, sex, smoking, BMI, waist circumference, hemoglobin, systolic blood pressure, LDL-cholesterol, HDL-cholesterol, triglyceride, blood glucose, uric acid, hypertension, cardiovascular disease, cancer, dyslipidemia, hyperuricemia, and prefecture. The association was clear negative monotonic risk increasing in those without proteinuria while the trends were less clear in those with proteinuria. P-for-interaction was detected as .01 for rapid CKD progression and .31 for initiation of KRT.

eGFR= estimated glomerular filtration rate, CKD=chronic kidney disease, KRT=kidney replacement therapy

© 2024 Ishimura N et al. *JAMA Health Forum*

eTable 1. Demographic characteristics of the study population by individual income levels

	Income deciles										
	**Overall**	**Decile 1 (Lowest)**	**Decile 2**	**Decile 3**	**Decile 4**	**Decile 5**	**Decile 6**	**Decile 7**	**Decile 8**	**Decile 9**	**Decile 10 (Highest)**
Number	5,591,060	682,814	480,004	617,011	715,976	350,331	690,841	570,585	568,919	468,880	445,699
Income, USD/year, mean (SD)	28,102 (19,659)	11,838 (2,000)	15,622 (702)	18,295 (864)	21,729 (867)	24,292 (0)	26,832 (865)	30,346 (867)	34,290 (1,301)	40,339 (2,139)	71,594 (44,051)
Age, mean (SD)	49.2 (9.3)	53.4 (10.0)	51.5 (9.7)	50.2 (9.8)	48.1 (9.4)	47.0 (8.9)	46.9 (8.7)	46.8 (8.1)	47.5 (7.8)	48.8 (7.7)	51.4 (8.5)
Sex, %, men	66.6	28.9	36.1	48.5	62.1	72.2	78.8	84.8	88.0	90.0	90.7
Smoking, %	34.4	21.8	26.2	30.2	35.3	38.3	39.6	40.8	40.4	39.2	34.6
BMI, kg/m^2, mean (SD)	23.3 (3.7)	22.7 (3.7)	22.7 (3.8)	22.9 (3.8)	23.2 (3.8)	23.4 (3.8)	23.5 (3.7)	23.6 (3.6)	23.7 (3.5)	23.8 (3.4)	24.0 (3.3)
Waist circumference, cm, mean (SD)	82.5 (9.9)	80.7 (10.0)	80.6 (10.1)	81.2 (10.2)	81.9 (10.2)	82.4 (10.1)	82.9 (9.9)	83.4 (9.7)	83.9 (9.5)	84.4 (9.2)	85.3 (8.9)
eGFR, $ml/min/1.73m^2$, mean (SD)	79.7 (14.2)	79.0 (15.0)	79.7 (14.8)	80.1 (14.7)	80.9 (14.4)	81.2 (14.1)	81.0 (14.0)	80.5 (13.7)	79.7 (13.5)	78.4 (13.3)	76.3 (13.3)
CKD Stage											
Stage 1 (eGFR [a] ≥90), %	21.7	21.3	22.4	22.9	24.1	24.5	23.8	22.6	20.5	17.9	14.3
Stage 2 (90> eGFR [a] ≥60), %	72.0	70.5	70.5	70.4	70.2	70.5	71.2	72.5	74.1	75.6	76.5
Stage 3 (60> eGFR [a] ≥30), %	6.2	8.0	7.0	6.6	5.5	4.9	5.0	4.8	5.3	6.4	9.1
Stage 4 (30> eGFR [a] ≥15), %	0.07	0.1	0.09	0.09	0.08	0.06	0.06	0.06	0.06	0.06	0.08
Stage 5 (15> eGFR [a]), %	0.06	0.1	0.06	0.07	0.06	0.06	0.06	0.06	0.05	0.06	0.05
Hb, g/dL, mean (SD)	14.5 (1.5)	13.6 (1.5)	13.8 (1.5)	14.1 (1.6)	14.4 (1.5)	14.6 (1.5)	14.8 (1.4)	14.9 (1.3)	15.0 (1.2)	15.0 (1.2)	15.0 (1.2)
Systolic BP, mmHg, mean (SD)	122.4 (16.9)	123.2 (18.1)	122.2 (17.9)	122.0 (17.6)	121.8 (17.1)	121.9 (16.7)	122.2 (16.5)	122.3 (16.3)	122.6 (16.1)	122.8 (16.0)	122.9 (15.9)
LDL-C, mg/dL, mean (SD)	124.3 (31.5)	123.5 (31.4)	122.7 (31.5)	122.7 (31.5)	123.3 (31.7)	124.0 (31.7)	124.5 (31.6)	125.2 (31.6)	125.8 (31.4)	126.2 (31.2)	126.0 (30.9)
HDL-C, mg/dL, mean (SD)	61.9 (16.5)	67.3 (17.0)	66.6 (17.0)	64.7 (16.8)	62.7 (16.6)	61.4 (16.3)	60.3 (16.1)	59.3 (15.7)	58.7 (15.5)	58.2 (15.2)	58.1 (15.1)
TG, mg/dL, mean (SD)	113.5 (85.6)	96.2 (66.3)	96.8 (68.5)	102.8 (76.4)	108.6 (82.7)	113.3 (87.0)	117.5 (89.1)	121.4 (92.1)	125.0 (93.6)	128.7 (94.7)	134.0 (96.7)
Glucose, mg/dL, mean (SD)	97.6 (19.0)	96.8 (18.3)	96.6 (18.5)	96.9 (18.8)	96.9 (18.9)	97.0 (19.0)	97.4 (19.0)	97.7 (18.9)	98.2 (19.0)	99.1 (19.2)	100.9 (19.8)
UA, mg/dL, mean (SD)	5.6 (1.4)	4.9 (1.3)	5.0 (1.3)	5.2 (1.4)	5.5 (1.4)	5.6 (1.4)	5.8 (1.4)	5.9 (1.3)	5.9 (1.3)	6.0 (1.3)	6.1 (1.3)
Comorbidity											
Hypertension, %	17.9	22.2	19.6	18.4	16.1	15.0	15.0	14.9	16.1	18.2	24.1
Diabetes, %	3.5	3.9	3.5	3.5	3.2	3.1	3.1	3.1	3.3	3.7	5.1
Cardiovascular disease, %	8.4	10.8	9.2	8.8	7.6	7.0	7.0	6.8	7.3	8.3	12.1
Cancer, %	2.6	4.0	3.4	3.1	2.5	2.1	2.0	1.8	1.9	2.1	3.1
Dyslipidemia, %	17.5	21.8	18.7	17.5	15.5	14.5	14.8	14.8	16.0	18.1	24.0
Hyperuricemia, %	5.2	3.7	3.6	4.1	4.4	4.6	4.9	5.3	5.9	6.9	9.6

a. $ml/min/1.73m^2$ USD=the United States dollar, SD=standard deviation, BMI=body mass index, eGFR=estimated glomerular filtration rate, Hb=hemoglobin, BP=blood pressure, LDL-C=low-density lipoprotein cholesterol, HDL-C=high-density lipoprotein cholesterol, TG=triglyceride and UA=uric acid

© 2024 Ishimura N et al. *JAMA Health Forum*

eTable 2. Demographic characteristics of the study population by individual income levels among men

	Income deciles										
	**Overall**	**Decile 1 (Lowest)**	**Decile 2**	**Decile 3**	**Decile 4**	**Decile 5**	**Decile 6**	**Decile 7**	**Decile 8**	**Decile 9**	**Decile 10 (Highest)**
Number	3,723,069	499,894	368,311	499,297	283,858	260,819	483,860	245,469	438,622	302,924	340,015
Income, USD/year, mean (SD)	31,893 (21,540)	14,477 (2,917)	20,021 (865)	23,435 (867)	26027 (0)	27,762 (0)	30,352 (867)	32,967 (0)	36,659 (1,284)	42,840 (1,997)	76,362 (46,859)
Age, mean (SD)	49.1 (9.5)	55.7 (10.7)	49.6 (10.4)	47.4 (9.6)	46.9 (9.1)	46.3 (8.5)	46.5 (8.2)	46.8 (7.7)	47.8 (7.7)	49.2 (7.6)	52.0 (8.6)
Smoking, %	43.2	38.8	45.3	46.3	45.9	46.0	45.4	44.5	43.2	41.2	36.3
BMI, kg/m^2, mean (SD)	23.9 (3.5)	23.7 (3.5)	23.7 (3.7)	23.8 (3.7)	23.9 (3.6)	23.8 (3.6)	23.9 (3.5)	23.9 (3.5)	24.0 (3.4)	24.1 (3.3)	24.3 (3.2)
Waist circumference, cm, mean (SD)	84.5 (9.4)	84.3 (9.5)	83.9 (9.9)	84.0 (9.8)	84.1 (9.7)	84.1 (9.6)	84.3 (9.4)	84.4 (9.3)	84.8 (9.1)	85.2 (8.8)	86.1 (8.5)
eGFR, ml/min/1.73m^2, mean (SD)	79.4 (14.2)	77.2 (15.4)	80.4 (14.8)	81.1 (14.3)	81.0 (14.1)	81.2 (13.9)	80.7 (13.7)	80.1 (13.5)	79.2 (13.4)	77.9 (13.3)	76.0 (13.3)
CKD Stage											
Stage 1 (eGFR [a] ≥90), %	20.1	18.5	23.8	24.6	24.2	24.1	22.9	21.3	19.4	16.8	13.8
Stage 2 (90> eGFR [a] ≥60), %	72.3	70.2	69.4	70.0	70.7	71.1	72.3	73.7	74.8	76.2	76.5
Stage 3 (60> eGFR [a] ≥30), %	6.6	11.0	6.6	5.3	5.1	4.7	4.7	4.9	5.7	6.8	9.5
Stage 4 (30> eGFR [a] ≥15), %	0.09	0.2	0.1	0.08	0.08	0.07	0.06	0.06	0.07	0.07	0.08
Stage 5 (15> eGFR [a]), %	0.08	0.2	0.1	0.08	0.06	0.07	0.06	0.06	0.06	0.06	0.05
Hb, g/dL, mean (SD)	15.2 (1.1)	15.0 (1.2)	15.1 (1.1)	15.2 (1.1)	15.2 (1.1)	15.2 (1.0)	15.2 (1.0)	15.2 (1.0)	15.2 (1.0)	15.2 (1.0)	15.2 (1.0)
Systolic BP, mmHg, mean (SD)	124.7 (16.3)	128.4 (17.4)	126.0 (16.8)	124.6 (16.4)	124.1 (16.2)	123.7 (16.0)	123.6 (16.0)	123.5 (15.9)	123.6 (15.8)	123.6 (15.8)	123.7 (15.5)
LDL-C, mg/dL, mean (SD)	125.5 (31.5)	123.5 (31.5)	124.7 (31.8)	125.3 (31.8)	125.4 (31.7)	125.6 (31.7)	126.0 (31.6)	126.2 (31.5)	126.3 (31.3)	126.5 (31.1)	126.1 (30.8)
HDL-C, mg/dL, mean (SD)	57.7 (15.0)	59.1 (15.8)	58.6 (15.5)	58.1 (15.2)	57.7 (15.0)	57.6 (15.0)	57.4 (14.8)	57.2 (14.7)	57.0 (14.6)	56.9 (14.5)	56.7 (14.3)
TG, mg/dL, mean (SD)	128.4 (95.0)	122.6 (88.4)	123.7 (92.7)	125.4 (94.4)	127.0 (95.2)	126.5 (94.2)	128.2 (96.0)	129.5 (96.5)	131.9 (97.2)	134.2 (97.0)	139.4 (99.3)
Glucose, mg/dL, mean (SD)	99.9 (20.5)	103.0 (22.7)	100.3 (21.5)	99.2 (20.6)	98.9 (20.1)	98.6 (19.9)	98.6 (19.6)	98.7 (19.4)	99.3 (19.6)	100.0 (19.6)	101.9 (20.3)
UA, mg/dL, mean (SD)	6.1 (1.2)	6.0 (1.3)	6.0 (1.3)	6.1 (1.2)	6.1 (1.2)	6.1 (1.2)	6.1 (1.2)	6.1 (1.2)	6.1 (1.2)	6.2 (1.2)	6.2 (1.2)
Comorbidity											
Hypertension, %	20.0	31.1	21.1	17.4	16.4	15.4	15.5	15.8	17.4	19.7	25.7
Diabetes, %	4.3	6.5	4.4	3.8	3.6	3.4	3.3	3.3	3.7	4.1	5.7
Cardiovascular disease, %	9.2	15.2	9.5	7.9	7.4	6.9	6.8	6.9	7.7	8.8	12.9
Cancer, %	2.2	4.0	2.3	1.8	1.7	1.5	1.5	1.5	1.7	2.0	3.1
Dyslipidemia, %	18.1	24.7	17.8	15.7	15.4	14.6	14.8	15.3	16.8	18.9	25.1
Hyperuricemia, %	7.4	9.4	6.9	6.3	6.2	6.0	6.1	6.4	7.0	8.0	10.9

a. ml/min/1.73m^2 USD=the United States dollar, SD=standard deviation, BMI=body mass index, eGFR=estimated glomerular filtration rate, Hb=hemoglobin, BP=blood pressure, LDL-C=low-density lipoprotein cholesterol, HDL-C=high-density lipoprotein cholesterol, TG=triglyceride and UA=uric acid

© 2024 Ishimura N et al. *JAMA Health Forum*

eTable 3. Demographic characteristics of the study population by individual income levels among women

	Income deciles										
	**Overall**	**Decile 1 (Lowest)**	**Decile 2**	**Decile 3**	**Decile 4**	**Decile 5**	**Decile 6**	**Decile 7**	**Decile 8**	**Decile 9**	**Decile 10 (Highest)**
Number	1,867,991	203,426	175,417	211,744	201,783	151,823	309,038	127,823	183,698	146,771	156,468
Income, USD/year, mean (SD)	20,546 (12,096)	10,293 (1,526)	12,719 (343)	14,311 (434)	16,033 (433)	17,351 (0)	19,890 (865)	22,556 (0)	25,105 (866)	29,266 (1,417)	46,130 (26,275)
Age, mean (SD)	49.5 (8.7)	52.4 (9.6)	51.1 (9.0)	50.2 (8.8)	49.4 (8.7)	49.2 (8.8)	48.0 (8.3)	47.9 (8.2)	47.9 (8.1)	48.2 (7.8)	50.8 (8.1)
Smoking, %	16.7	15.6	16.8	17.6	17.9	17.6	18.0	17.3	16.4	15.4	13.3
BMI, kg/m^2, mean (SD)	22.2 (3.8)	22.5 (3.8)	22.3 (3.8)	22.3 (3.8)	22.2 (3.8)	22.2 (3.8)	22.2 (3.8)	22.2 (3.8)	22.2 (3.8)	22.2 (3.7)	22.1 (3.6)
Waist circumference, cm, mean (SD)	78.7 (9.8)	79.5 (9.9)	79.0 (9.9)	78.7 (9.9)	78.6 (9.9)	78.6 (9.8)	78.4 (9.9)	78.4 (9.8)	78.4 (9.8)	78.5 (9.8)	78.8 (9.6)
eGFR, ml/min/1.73m^2, mean (SD)	80.4 (14.3)	80.0 (14.8)	80.6 (14.6)	80.7 (14.5)	80.8 (14.4)	80.6 (14.4)	81.0 (14.3)	80.8 (14.2)	80.4 (14.0)	79.9 (13.9)	78.3 (13.7)
CKD Stage											
Stage 1 (eGFR [a] ≥90), %	23.0	22.8	23.8	23.9	23.9	23.6	24.0	23.6	22.8	21.6	18.4
Stage 2 (90> eGFR [a] ≥60), %	71.4	70.6	70.5	70.6	70.8	70.8	71.0	71.4	72.0	72.9	74.6
Stage 3 (60> eGFR [a] ≥30), %	5.5	6.5	5.6	5.4	5.2	5.6	4.9	5.0	5.1	5.5	6.9
Stage 4 (30> eGFR [a] ≥15), %	0.05	0.07	0.05	0.05	0.05	0.03	0.04	0.04	0.03	0.03	0.04
Stage 5 (15> eGFR [a]), %	0.03	0.06	0.04	0.05	0.02	0.02	0.02	0.02	0.02	0.02	0.02
Hb, g/dL, mean (SD)	13.1 (1.3)	13.1 (1.3)	13.1 (1.3)	13.1 (1.3)	13.1 (1.3)	13.1 (1.3)	13.1 (1.3)	13.1 (1.3)	13.1 (1.2)	13.1 (1.2)	13.2 (1.2)
Systolic BP, mmHg, mean (SD)	117.8 (17.0)	121.6 (17.9)	120.5 (17.6)	119.5 (17.4)	118.5 (17.2)	117.7 (16.9)	116.7 (16.5)	116.0 (16.4)	115.7 (16.3)	115.3 (16.1)	115.5 (16.3)
LDL-C, mg/dL, mean (SD)	122.0 (31.4)	124.4 (31.6)	123.5 (31.5)	122.6 (31.4)	122.1 (31.4)	121.7 (31.3)	120.8 (31.2)	120.5 (31.1)	120.6 (31.1)	121.0 (31.1)	123.3 (31.6)
HDL-C, mg/dL, mean (SD)	70.4 (16.1)	70.4 (16.4)	70.7 (16.4)	70.8 (16.3)	70.5 (16.2)	70.3 (16.1)	70.2 (16.0)	70.1 (15.9)	70.1 (16.0)	70.2 (15.9)	70.6 (16.1)
TG, mg/dL, mean (SD)	83.8 (51.1)	86.9 (52.5)	84.4 (51.0)	83.3 (50.1)	83.0 (50.6)	83.6 (50.9)	82.3 (50.0)	82.6 (51.7)	82.6 (51.0)	83.2 (51.3)	86.4 (52.9)
Glucose, mg/dL, mean (SD)	93.1 (14.3)	94.4 (15.3)	93.8 (14.9)	93.4 (14.6)	93.0 (14.4)	92.8 (14.2)	92.5 (13.9)	92.4 (13.5)	92.4 (13.6)	92.6 (13.8)	93.3 (13.7)
UA, mg/dL, mean (SD)	4.5 (1.0)	4.5 (1.0)	4.5 (1.0)	4.5 (1.0)	4.5 (1.0)	4.5 (1.0)	4.5 (1.0)	4.5 (1.0)	4.5 (1.0)	4.5 (1.0)	4.6 (1.0)
Comorbidity											
Hypertension, %	13.7	18.7	16.3	14.7	13.5	13.2	11.8	11.4	11.5	11.5	13.9
Diabetes, %	2.0	2.8	2.3	2.1	2.0	2.0	1.7	1.8	1.7	1.8	2.2
Cardiovascular disease, %	6.9	8.9	7.5	6.7	6.5	6.7	6.0	6.2	6.2	6.4	8.2
Cancer, %	3.5	3.8	3.4	3.3	3.3	3.3	3.2	3.4	3.4	3.7	4.3
Dyslipidemia, %	16.1	21.4	18.5	16.9	15.7	15.7	13.8	13.8	13.7	14.1	17.8
Hyperuricemia, %	0.7	1.0	0.8	0.7	0.7	0.7	0.6	0.7	0.6	0.6	0.9

a. ml/min/1.73m^2 USD=the United States dollar, SD=standard deviation, BMI=body mass index, eGFR=estimated glomerular filtration rate, Hb=hemoglobin, BP=blood pressure, LDL-C=low-density lipoprotein cholesterol, HDL-C=high-density lipoprotein cholesterol, TG=triglyceride and UA=uric acid

© 2024 Ishimura N et al. *JAMA Health Forum*

eTable 4. Demographic characteristics of the study population undertaking health check-ups and not undertaking health check-ups

	**Individuals undertaking health check-ups in fiscal year 2015 (Study population)**	**Missing, N (%)**	**Individuals not undertaking health check-ups in fiscal year 2015 (Excluded population)**	**Missing, N (%)**	***P*-value [b]**
Number	7,512,083	N/A	10,478,597	N/A	
Income, USD/year, mean (SD)	27,431 (19,427)	0 (0)	25,510 (25,441)	17 (< .01)	< .001
Age [a], mean (SD)	49.0 (9.6)	0 (0)	50.0 (10.4)	0 (0)	< .001
Sex, %, men	65.1	0 (0)	60.8	0 (0)	< .001

a. age in April,2015

b. P-value was calculated by independent sample's t-test and Pearson's chi-square test

© 2024 Ishimura N et al. *JAMA Health Forum*

eTable 5. Results of the proportional hazard tests based on Schoenfeld residuals between income deciles

	***P*-value**		***P*-value**
Income Decile 1	.95	Income Decile 6	.71
Income Decile 2	.35	Income Decile 7	.56
Income Decile 3	.12	Income Decile 8	.70
Income Decile 4	.70	Income Decile 9	.95
Income Decile 5	.64	Income Decile 10	Ref

© 2024 Ishimura N et al. *JAMA Health Forum*

eTable 6. Slope and relative index of inequalities in our study population

	SII (95%CI)	RII (95%CI)	KMI (95%CI)
**(i) rapid CKD progression (cases/10,000 persons)**	349.5 (342.5 to 356.4)	0.60 (0.59 to 0.62)	1.87 (1.84 to 1.89)
**(ii) KRT initiation (cases/million person-years)**	95.2 (72.6 to 117.8)	0.38 (0.30 to 0.47)	1.48 (1.34 to 1.61)

SII=slope index of inequality, RII=relative index of inequality, KMI=Kunst Mackenbach relative index, eGFR=estimated glomerular filtration rate, CKD=chronic kidney disease, KRT=kidney replacement therapy and CI=confidence interval

© 2024 Ishimura N et al. *JAMA Health Forum*

eTable 7. Absolute risk difference for rapid CKD progression between the 1st to 9th decile and the 10th decile

	**Absolute risk difference for rapid CKD progression (cases/10,000 persons) (vs. 10th decile) (95%CI)**
Income Decile 1	+301.2 (291.9 to 310.5)
Income Decile 2	+265.3 (255.5 to 275.2)
Income Decile 3	+229.8 (220.6 to 239.0)
Income Decile 4	+186.4 (177.6 to 195.2)
Income Decile 5	+147.7 (137.3 to 158.1)
Income Decile 6	+103.0 (94.2 to 111.8)
Income Decile 7	+67.4 (58.2 to 76.5)
Income Decile 8	+39.0 (29.9 to 48.2)
Income Decile 9	+22.1 (12.6 to 31.7)

CKD=chronic kidney disease and CI=confidence interval

© 2024 Ishimura N et al. *JAMA Health Forum*

eTable 8. Population attributable risks of rapid CKD progression and KRT initiation by setting income levels at top 10th and 50th percentiles

1. **Unexposed population: the highest income group** (top 10th percentile)	**Relative risk for exposed population (95%CI)**	**PAR [a,b]** Reduction of the percentage of cases	**PAR [a,b]** Reduction of the number of cases
(i) rapid CKD progression	OR 1.32 (1.30 to 1.34)	22.6 %	73,112
(ii) KRT initiation	HR 1.40 (1.27 to 1.54)	26.4 %	1,568
2. **Unexposed population: people above the median** (top 50th percentile)	**Relative risk for exposed population (95%CI)**	**PAR [a,b]** Reduction of the percentage of cases	**PAR [a,b]** Reduction of the number of cases
(i) rapid CKD progression	OR 1.37 (1.36 to 1.38)	15.5 %	50,225
(ii) KRT initiation	HR 1.22 (1.15 to 1.28)	9.8 %	581

a. PAR (%) =Pe(OR-1)/Pe(OR-1)+1, Pe= the proportion of exposure in the population and OR= odds ratio

b. PAR (%) =Pe(HR-1)/Pe(HR-1)+1, Pe= the proportion of exposure in the population and HR= hazard ratio

CKD=chronic kidney disease, KRT=kidney replacement therapy, PAR=population attributable risk and CI=confidence interval

© 2024 Ishimura N et al. *JAMA Health Forum*
