# Supplementary material for: Income Level and Impaired Kidney Function Among Working Adults in Japan
Source: JAMA Health Forum. 2024 Mar 1;5(3):e235445. doi: 10.1001/jamahealthforum.2023.5445 (PMC10907921; doi:10.1001/jamahealthforum.2023.5445)
Supplement: Supplement 2. — Data Sharing Statement [file jamahealthforum-e235445-s002.pdf]

Data Sharing Statement

Ishimura. Income Level and Impaired Kidney Function Among Working Adults in Japan. *JAMA Health Forum*. Published March 01, 2024. doi:10.1001/jamahealthforum.2023.5445

Data
Data available: No

Additional Information
Explanation for why data not available: The data that support the findings of this study are available from the Japan Health Insurance Association; however, we used these under license for the current study, and so the data are not publicly available.
